# Isoflurane preconditioning protects against renal ischemia/reperfusion injury in diabetes via activation of the Brg1/Nrf2/HO-1 signaling pathway

**DOI:** 10.1590/acb396124

**Published:** 2024-09-30

**Authors:** Daojing Gong, Ziqiang Dong, Xiaobo Chen, Hao Chen, Huihuang Lin

**Affiliations:** 1 China Three Gorges University – The First Clinical College – Department of Urology – Yichang – China.

**Keywords:** Isoflurane, Diabetes Mellitus, Ischemia, Reperfusion, Kidney

## Abstract

**Purpose:**

To examine whether isoflurane preconditioning (IsoP) has a protective effect against renal ischemia/reperfusion injury (I/RI) in diabetic conditions and to further clarify the underlying mechanisms.

**Methods:**

Control and streptozotocin-induced diabetic rats were randomly assigned to five groups, as follows: normal sham, normal I/R, diabetic sham, diabetic I/R, and diabetic I/R + isoflurane. Renal I/RI was induced by clamping renal pedicle for 45 min followed by reperfusion for 24 h. IsoP was achieved by exposing the rats to 2% isoflurane for 30 min before vascular occlusion. Kidneys and blood were collected after reperfusion for further analysis. Renal histology, blood urea nitrogen, serum creatinine, oxidative stress, inflammatory cytokines, and renal cell apoptosis were assessed. Furthermore, the expression of brahma related gene 1 (Brg1), nuclear factor-erythroid 2-related factor 2 (Nrf2), heme oxygenase-1 (HO-1), and nuclear factor-κB (NF-κB) were determined.

**Results:**

Compared with control, diabetic rats undergoing I/R presented more severe renal injury, oxidative stress, inflammatory reaction, and apoptosis with the impairment of Brg1/Nrf2/HO-1 signaling. All these alterations were significantly attenuated by pretreatment with isoflurane.

**Conclusions:**

These findings suggest that isoflurane could alleviate renal I/RI in diabetes, possibly through improving Brg1/Nrf2/HO-1 signaling.

## Introduction

Ischemia/reperfusion (I/R) injury is defined as tissue damage resulting from blood flow restoration to the tissue following a period of ischemia. Renal I/R injury (RI/RI) is a major cause of acute postoperative renal dysfunction and renal failure, which can occur in many clinical settings, including kidney transplantation and partial nephrectomy, leading to a series of complications, such as delayed graft function, graft rejection, and acute kidney injury^1^^,^ 2. The underlying mechanisms of RI/RI have bene widely studied, and oxidative stress and the inflammatory response have been reported to be crucial in the initiation of RI/RI. However, no effective means are currently available to prevent RI/RI. Diabetes mellitus is the leading cause of chronic kidney disease in most developed countries^3^. Clinically, patients suffering from RI/RI have a poor prognosis in terms of serious morbidity and mortality. Furthermore, animal model experiments have confirmed that diabetic rats are more susceptible to RI/RI than normal rats^4^^,^ 5; therefore, it is of great clinical significance to prevent RI/RI in patients with diabetes.

Isoflurane is a halogenated inhalational anesthetic commonly used in clinical practice. Previous studies have revealed that, in addition to its anesthetic effect, isoflurane exerts a protective role during I/R injury in some tissues and organs including the brain^6^, heart^7^, lung^8^, and liver^9^. Furthermore, isoflurane has been reported to ameliorate RI/RI in a preconditioning manner via anti-inflammation and anti-apoptosis effects^10^. Furthermore, research has shown that isoflurane preconditioning (IsoP) has a protective effect in RI/RI, which is mediated in part through the reduction in oxidative stress^11^; however, the mechanisms by which IsoP protects against RI/RI in diabetes remain unknown.

Brahma-related gene 1 (Brg1) is a core catalytic subunit of the SWI/SNF chromatin-remodeling complex and plays a key role in gene activation and transcription in mammalian cells^12^. An increasing number of studies have reported that Brg1 interacts with a variety of nuclear proteins, including transcription factors, nuclear receptors, and chromatin-modifying enzymes, and is involved in multiple biological processes, such as cell proliferation, differentiation, and apoptosis^13^^–^ 16. Interestingly, Brg1 has been showed to counteract hepatic I/R injury and neuronal hypoxia/reoxygenation injury through the suppression of oxidative stress and apoptosis^17^^,^ 18. Furthermore, a previous study has demonstrated that Brg1 facilitates Z-DNA formation and the subsequent recruitment of RNA polymerase II, which is critical in nuclear factor-erythroid 2-related factor 2 (Nrf2) activation and its downstream antioxidant gene expression^19^. However, whether Brg1 is involved in the regulation of RI/RI and oxidative stress remains unclear.

Considering the rapidly increasing incidence of diabetes, further research is required to explore effective interventions against RI/RI in patients with diabetes. By establishing an RI/RI model in streptozotocin (STZ)-induced diabetic rats, we attempted to test the following hypothesis: IsoP reduces oxidative stress, inflammation, and apoptosis via activation of the Brg1/Nrf2 signaling pathway in the context of diabetes, thereby mitigating RI/RI.

## Methods

### Animals and induction of diabetes

Male Sprague-Dawley rats, weighing 180–200 g, were purchased from the Experimental Animal Center of China Three Gorges University (Yichang, China). The animal study protocol was approved by the Animal Experiment Ethics Committee of China Three Gorges University, and the procedures were performed in adherence with the Guidelines of the Care and Use of Laboratory Animals. All rats were housed in a temperature and humidity-controlled environment (22–24°C; relative humidity 50–70%) with a 12 h light/dark cycle and free access to food and water.

Rats were randomly divided into the following five groups (n = 8 per group):

- Normal sham-operated group (NS);
- Normal I/R group (NI/R);
- Diabetic sham-operated group (DS);
- Diabetic I/R group (DI/R);
- IsoP + diabetic I/R group (Iso + DI/R).

Diabetes was induced via intraperitoneal injection of STZ (Sigma–Aldrich, St. Louis, MO, United States of America) solution dissolved in citrate buffer (0.1 mM, pH 4.5) at a single dose of 65 mg/kg, as described previously^20^. The control rats were administered an equal volume of citrate buffer via intraperitoneal injection. Three days after STZ administration, tail vein blood was collected to check the glycemic level with a glucometer (Terumo, Tokyo, Japan). Only rats with a random blood sugar concentration > 16.7 mM for three consecutive measurements were considered to be diabetic. All animals were provided with a plentiful normal diet and water ad libitum and raised for another eight weeks before renal surgery.

### Isoflurane preconditioning and renal ischemia/reperfusion injury

Rats were placed in the induction chamber of a portable anesthesia instrument (cat. no. AS-01-0007; Summit Anesthesia Solutions, Bend, OR, United States of America) before undergoing RI/RI.

Once the isoflurane (Lunan Better Pharmaceutical Co., Ltd., Shandong, China) concentration was stabilized at 2% (airflow: 1 L/min), rats in the Iso + DI/R group were allowed to inhale isoflurane for 30 min, followed by 15 min of discharge before RI/RI. Rats in other groups inhaled only 100% oxygen for 30 min.

Body weight and blood glucose were measured before the I/R operation. Animals were anesthetized by intraperitoneal injection of pentobarbital sodium (60 mg/kg) and then subjected to an abdominal midline incision. After kidney exposure, right nephrectomy was performed, and the left renal pedicle was clamped for 45 min using a non-trauma vascular clamp, which was followed by a clamp removal for 24 h of reperfusion. The same procedure was performed in sham-operated animals without renal pedicle occlusion. All rats were sacrificed by cervical dislocation after 24 h of reperfusion. Plasma samples were harvested for renal function tests. The left kidney was removed and split coronally into two pieces for subsequent analyses. One piece was fixed in 10% phosphate-buffered formalin, and the other was immediately stored at -80ºC.

### Histopathological examination

After embedding in paraffin, 4-μm-thick sections were prepared for hematoxylin and eosin (H&E) staining. Morphological changes were observed in a blinded manner by two experienced renal pathologists. The degree of tubular damage was histologically graded from 0 to 4, according to the following criteria^21^:

- 0: no obvious tubular necrosis;
- 1: individual proximal convoluted tubule cell necrosis;
- 2: necrosis involving all the adjacent proximal convoluted tubule cells with survival of the surrounding renal tubules;
- 3: necrosis limited to the distal third of the proximal convoluted tubule with the inner cortex affected;
- 4: necrosis spreading to all three proximal convoluted tubule segments.

Ten separate fields were randomly selected for each slide to calculate the tubular injury score using an optical microscope (magnification, ×400, Olympus Corporation, Tokyo, Japan).

### Assessment of renal function

Whole blood was centrifuged at 2,000 × g for 15 min at 4°C to obtain serum. Blood urea nitrogen (BUN) and serum creatinine (Scr) were measured using an automatic biochemistry analyzer (Hitachi 7060, Tokyo, Japan).

### Superoxide dismutase and malondialdehyde measurement

Superoxide dismutase (SOD) is an important enzyme in oxidative stress, and malondialdehyde (MDA) is a terminal product of lipid peroxidation. The washed kidney tissues were cut into fragments and homogenized on ice with a glass homogenizer. The homogenates were centrifuged at 12,000 × g for 10 min, and the supernatants were collected. The MDA content and SOD activity in renal tissues were measured using commercial assays (Jiancheng Bioengineering Institute, Nanjing, China), according to the manufacturer’s protocols.

### Enzyme-linked immunosorbent assay

The levels of tumor necrosis factor (TNF)-α and interleukin (IL)-1β in the homogenized renal tissues were determined by commercial enzyme-linked immunosorbent assay (ELISA) (Elabscience Biotechnology Co., Ltd, Wuhan, China), according to the manufacturer’s instructions. The plate was read at 450 nm.

### Terminal deoxynucleotidyl-transferase-mediated dUTP nick-end labeling

Kidney tissue apoptosis was detected with an In Situ Cell Death Detection Kit (Roche Diagnostics GmbH, Mannheim, Germany), according to the manufacturer’s instruction. Briefly, 4-μm-thick paraffin-embedded sections underwent routine deparaffinization and rehydration, followed by treatment with proteinase K solution (20 μg/mL) at 37°C for 15 min. Subsequently, the slides were washed with phosphate buffered saline (PBS) followed by the addition of the terminal deoxynucleotidyl-transferase-mediated dUTP nick-end labeling (TUNEL) reaction mixture for 60 min at 37°C. Sections were then washed with PBS, and the cell nuclei were displayed after staining with 4’,6-diamidino-2-phenylindole (DAPI) in the dark. Finally, sections were analyzed, and the apoptosis index was calculated from five random fields per section according Eq. 1.


Apoptotic cells/Total cells×100% (1)

### Western blotting

Cytosolic and nuclear proteins were extracted from renal tissues using a Nuclear and Cytoplasmic Protein Extraction Kit (NanJing KeyGen Biotech Co., Ltd., Nanjing, China), according to the manufacturer’s protocol. The protein concentration was determined by the bicinchoninic acid (BCA) method. Equal amounts of protein (40 μg/lane) were separated by 10% sodium dodecyl sulfate-polyacrylamide gel electrophoresis (SDS-PAGE) and then transferred to polyvinylidene fluoride (PVDF) membranes. Each membrane was blocked with tris buffered saline with tween (TBST) containing 5% non-fat milk at room temperature for 1 h. Subsequently, membranes were incubated overnight at 4ºC with primary antibodies against Brg1 (cat. no. ab207612; 1:1,000; Abcam, Cambridge, United Kingdom), Nrf2 (cat. no. 80593-1-RR; 1:1,000), heme oxygenase-1 (HO-1; cat. no. 10701-1-AP; 1:1,000), B-cell lymphoma-2 (Bcl-2; cat. no. 12789-1-AP; 1:1,000), Bcl-2-associated X protein (Bax; cat. no. 50599-2-IG; 1:1,000), lamin B (cat. no. 66095-1-IG; 1:5,000), β-actin (cat. no. 66009-1-IG; 1:5,000; all Proteintech Group, Inc., Rosemont, IL, United States of America), nuclear factor-κB (NF-κB) p65 (cat. no. #8242; 1:1,000), and cleaved caspase-3 (cat. no. #9664; 1:1,000; all Cell Signaling Technology, Danvers, MA, United States of America), followed by incubation with a secondary goat-anti-mouse or goat-anti-rabbit horseradish peroxidase-conjugated antibody (both 1:5,000) at room temperature for 1 h. Bands were visualized using the equivalent mixture of enhanced luminol reagents and oxidizing reagents (Thermo Fisher Scientific), and band densities were quantitated using Glyko BandScan gel analysis software (Glyko, Novato, CA, United States of America).

### Statistical analysis

Data were expressed as mean ± standard deviation (SD). One-way analysis of variance was used to determine significant differences among the five groups. Differences between two groups were analyzed using the Student–Newman–Keuls’ test. *p* < 0.05 was considered significantly different. All statistical analyses were performed using GraphPad Prism software version 7.0 (GraphPad Software, Inc., San Diego, CA, United States of America).

## Results

### Characteristics of diabetic and control rats

As shown in Table 1, eight weeks after STZ administration, diabetic rats displayed typical diabetic features including hyperglycemia, weight loss, polydipsia, and polyphagia. Compared with normal rats, the blood sugar level in diabetic rats was significantly increased, and their body weight was remarkably reduced.

**Table 1:** General characteristics of normal and diabetic rats after eight weeks*.

Characteristics	NS (n = 8)	NI/R (n = 8)	DS (n = 8)	DI/R (n = 8)	Iso + DI/R (n = 8)
Random glycemic level (mM)	6.5 ± 0.6	5.9 ± 0.8	22.6 ± 1.5*	23.6 ± 2.2*	23.2 ± 2.0*
Body weight (g)	427.6 ± 8.4	435.1 ± 7.6	290.2 ± 9.8*	301.1 ± 10.5*	292.5 ± 10.1*

* The data are expressed as mean ± standard deviation;

NS: normal sham-operated group; NI/R: normal ischemia/reperfusion (I/R) group; DS: diabetic sham-operated group; DI/R: diabetic I/R group; Iso: isoflurane;

** *p* < 0.05 *versus* NS group and NI/R group.

Source: Elaborated by the authors.

### Isoflurane preconditioning alleviates renal ischemia/reperfusion injury in diabetic rats

**Figure 1:**
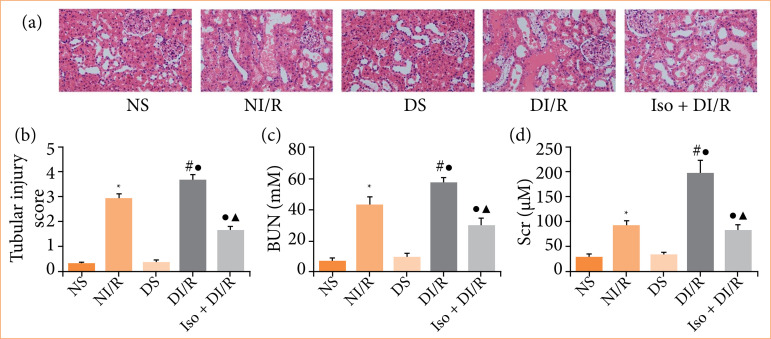
Renal ischemia/reperfusion (I/R) injury after 45 min of ischemia followed by 24 h of reperfusion. **(a)** Representative photomicrographs of renal tissues (magnification, ×400). **(b)** Renal injury score. **(c)** Serum BUN concentration. **(d)** Serum Scr concentration. The data are expressed as mean ± standard deviation.

NS: normal sham-operated group; NI/R: normal I/R group; DS: diabetic sham-operated group; DI/R: diabetic I/R group; Iso: isoflurane; BUN: blood urea nitrogen; Scr: serum creatinine; *p < 0.05 *versus* NS group; #*p* < 0.05 *versus* NI/R group; ●*p* < 0.05 *versus* DS group; ▲*p* < 0.05 *versus* DI/R group. Source: Elaborated by the authors.

The degree of renal tubular damage in each group is shown in Figs. 1a and 1b. Tubular dilatation, loss of the brush border, cast formation, and cell lysis were the histopathological changes observed in the kidney tubules following RI/RI. Kidneys in the diabetic group exhibited more serious tubular damage (*p* < 0.05). The trends in the BUN and Scr levels were consistent with the morphological findings (Figs. 1c and 1d). However, IsoP prevented RI/RI in diabetic rats, as demonstrated by an obvious decrease in microscopic damage scores and BUN and Scr levels compared with the DI/R group (*p* < 0.05).

### Isoflurane preconditioning alleviates renal ischemia/reperfusion injury-induced oxidative stress and inflammation in diabetic rats

**Figure 2:**
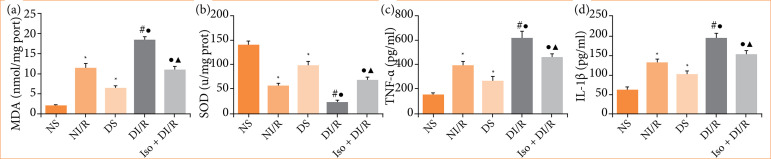
Levels of oxidative stress and inflammation in renal tissues. **(a)** MDA concentration. **(b)** SOD activity. **(c)** TNF-α level. **(d)** IL-1β level.

NS: normal sham-operated group; NI/R: normal I/R group; DS: diabetic sham-operated group; DI/R: diabetic I/R group; Iso: isoflurane; MDA: malondialdehyde; SOD: superoxide dismutase; TNF: tumor necrosis factor; IL: interleukin; *p < 0.05 versus NS group; #*p* < 0.05 *versus* NI/R group; ●*p* < 0.05 *versus* DS group; ▲*p* < 0.05 *versus* DI/R group. Source: Elaborated by the authors.

As shown in Figs. 2a and 2b, we observed a significant decrease in SOD activity combined with a significant increase in the MDA content in the NI/R and DI/R groups, as compared to the NS and DS groups, respectively (*p* < 0.05). The oxidative abnormalities were further aggravated in the DI/R group compared to the NI/R group (*p* < 0.05). In addition, I/R induced significant increases in the levels of TNF-α and IL-1β in both normal and diabetic rats, with the most remarkable increase observed in the DI/R group (Figs. 2c and 2d, *p* < 0.05). However, IsoP relieved oxidative abnormalities, as indicated by a significant increase in SOD activity and a decrease in the MDA content compared to the DI/R group (*p* < 0.05). Meanwhile, IsoP reduced TNF-α and IL-1β levels compared to the DI/R group (*p* < 0.05).

### Isoflurane preconditioning inhibits renal ischemia/reperfusion injury-induced apoptosis in diabetic rats

**Figure 3:**
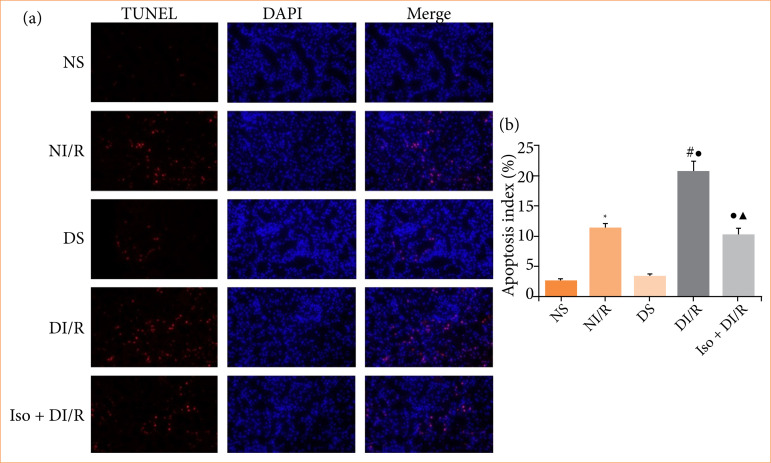
Renal cell apoptosis detected by TUNEL staining. **(a)** Representative fluorescence microscopy images (magnification, ×400) and **(b)** quantitative analysis of TUNEL staining.

NS: normal sham-operated group; NI/R: normal I/R group; DS: diabetic sham-operated group; DI/R: diabetic I/R group; Iso: isoflurane; TUNEL: terminal deoxynucleotidyl-transferase-mediated dUTP nick-end labeling; DAPI: 4,6-diamino-2-phenyl indole; *p < 0.05 versus NS group; #p < 0.05 versus NI/R group; ●p < 0.05 versus DS group; ▲p < 0.05 versus DI/R group. Source: Elaborated by the authors.

**Figure 4:**
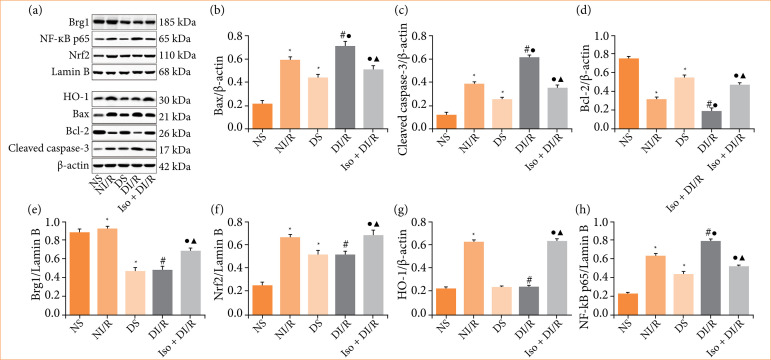
Kidney protein expression of Bax, cleaved caspase-3, Bcl-2, Brg1, Nrf2, HO-1 and NF-κB after renal ischemia/reperfusion injury detected by western blotting. **(a)** Representative western blotting and (b–h) quantitation of western blotting data.

NS: normal sham-operated group; NI/R: normal I/R group; DS: diabetic sham-operated group; DI/R: diabetic I/R group; Iso: isoflurane; Bcl-2: B-cell lymphoma-2; Brg1: brahma related gene 1; HO-1: heme oxygenase-1; NF-κB: nuclear factor-κB; *p < 0.05 *versus* NS group; #*p* < 0.05 *versus* NI/R group; ●*p* < 0.05 *versus* DS group; ▲*p* < 0.05 *versus* DI/R group. Source: Elaborated by the authors.

The TUNEL assay was performed to estimate apoptosis in renal tubular epithelial cells (Figs. 3a and 3b). The number of TUNEL-positive (apoptotic) cells significantly increased after I/R in both the normal and diabetic groups, with the most significant increase observed in the DI/R group (*p* < 0.05). Apoptosis-related protein expression in renal tissues was detected by western blotting (Figs. 4a–4d). Bax and cleaved caspase-3 levels increased after I/R in both the normal and diabetic rats, while the greatest increase in both proteins was observed in the DI/R group (*p* < 0.05). Bcl-2 expression was decreased in both the NI/R and DI/R groups compared to their corresponding sham groups, while decreased Bcl-2 expression in the DI/R group was the most remarkable (*p* < 0.05). However, compared to the DI/R group, IsoP significantly reduced apoptosis (*p* < 0.05). Consistently, IsoP markedly decreased the expression of Bax and cleaved caspase-3, and increased Bcl-2 expression, compared to the DI/R group (*p* < 0.05).

### Isoflurane preconditioning activates the Brg1/Nrf2/HO-1 pathway after ischemia/reperfusion stimulation in diabetic rats

As shown in Figs. 4a and 4e–4h, Brg1 expression was lower in both the DS and DI/R group compared to their corresponding control groups (NS group and NI/R group), respectively (*p* < 0.05). Likewise, although the nuclear level of Nrf2 was higher in the DS group compared to the NS group (*p* < 0.05), HO-1 expression levels were similar between the NS and DS groups (*p* > 0.05). After I/R stimulation, with the further increase of nuclear NF-κB levels, the expression of both Nrf2 and HO-1 was unchanged in the DI/R group compared to the DS group (*p* > 0.05). However, IsoP elevated the levels of Brg1 and Nrf2, as well as the downstream HO-1 expression, compared with the DI/R group (*p* < 0.05). Furthermore, IsoP significantly inhibited the increase in NF-κB in diabetic rats undergoing RI/RI compared to the DI/R group (*p* < 0.05).

## Discussion

Our results in an STZ-induced diabetic rat model demonstrate that IsoP prior to I/R can significantly alleviate renal injury in terms of histological improvements, decreased BUN and Scr concentrations, and reduced tubular cells apoptosis. Furthermore, IsoP can enhance the antioxidant capacity and modulate the inflammatory response through activation of the Brg1/Nrf2/HO-1 signaling pathway after RI/RI, even in the setting of hyperglycemia.

At present, there are few studies on RI/RI in diabetic rats, both at home and abroad. Previous studies have shown that the mechanism of RI/RI involves many factors; however, the mechanism is not completely clear, although may be closely related to the production of excessive oxygen free radicals, intracellular calcium overload, mitochondrial dysfunction, and inflammation^22^^,^ 23. Various inflammatory mediators generated during I/R injury may interact with apoptosis, thereby gradually amplifying the apoptotic signal transduction pathway, thus making the mechanism of RI/RI more complex.

A previous study revealed that diabetic rats undergoing renal I/R have higher levels of apoptosis, BUN, and Scr, and that the increased susceptibility to I/R in diabetic rats may correlate to the overexpression of inflammatory factors^5^. Recent studies have shown that the increased diabetic kidney I/R sensitivity is also related to oxidative stress^20^^,^ 24. Consistent with this, we found that, compared to the NI/R group, the DI/R group had higher BUN and Scr levels and renal tubular pathological scores. In addition, the SOD activity was decreased in both the NI/R and DI/R groups, especially in the DI/R group, and the MDA content was increased in both the NI/R and DI/R groups, especially in the DI/R group. Furthermore, compared to the NI/R group, the expression of TNF-α and IL-1β was increased, as were the levels of cell apoptosis, in the renal tissues of diabetic rats undergoing I/R. These results indicate that diabetes exacerbates RI/RI through the enhancement of oxidative stress, the inflammatory response, and apoptosis following the I/R insult.

Nrf2 is a key redox-sensitive transcription factor that mainly promotes the expression of antioxidant genes, including HO-1, and resists oxidative damage^25^^–^ 27. Normally, Nrf2 is anchored in the cytoplasm by Kelch-like ECH-associated protein 1 (KEAP1) and is degraded through ubiquitination^28^. When oxidative stress occurs, Nrf2 dissociates from KEAP1, enters the nucleus, and binds to the antioxidant response element (ARE). Brg1, which is a key component of the SWI/SNF chromatin-remodeling complex, directly mediates structural changes in chromatin to facilitate transcription factors binding to DNA, promoting gene transcription^29^. Zhang et al.^19^ have revealed that Brg1 contributes to the formation of Z-DNA and the recruitment of RNA polymerase II, which is crucial for Nrf2-mediated induction of HO-1 expression. In addition, Brg1 has been reported to inhibit oxidative stress and protect against hepatic and cerebral I/R injury via activation of the Nrf2-mediated antioxidant reaction^17^^,^ 18. However, the Brg1/Nrf2 signaling-mediated protective effect against myocardial I/R injury is blocked under a high glucose environment^29^^,^ 30. Our previous research has confirmed that impairment of the Nrf2 antioxidant capacity is responsible for the aggravation of RI/RI in diabetes^20^. In this study, we further confirmed that the reduction of Nrf2 activity in the renal tissue of diabetic rats is associated with the inhibition of Brg1 expression, which is consistent with the mentioned studies. This suggests that increased susceptibility to RI/RI in diabetic rats is relevant to the suppression of Brg1/Nrf2/HO-1 signaling in the kidney.

Isoflurane is an inhaled anesthetic, which is commonly applied for the induction and maintenance of general anesthesia. In addition to its anesthetic effect, isoflurane exerts organ-protective effects against I/R injury. Liang et al.^10^ demonstrated that IsoP can protect against RI/RI through the inhibition of NF-κB-mediated inflammation and a decrease in tubular apoptosis in rats. Previous studies have shown that isoflurane can dilate coronary arteries and produce coronary steal, leading to myocardial ischemia. However, these studies have shortcomings, such as insufficient number of patients studied and lack of hemodynamic control during the study period. Leung et al.^31^ demonstrated that anesthetics, including isoflurane, may have a protective effect on the myocardium at risk under strict hemodynamic control. Ran et al.^32^ have shown that IsoP can inhibit the expression of TNF-α and capase-3 in the rabbit myocardium, thus playing a protective role in rabbit myocardial I/R injury. Isoflurane also has a protective effect on intestinal I/R injury, mainly through the activation of PPAR-γ, which further inhibits the expression of TNF-α and other inflammatory factors^33^. The application of isoflurane anesthesia can suppress TNF-α-related inflammatory cytokines and oxidative stress in a rat liver I/R injury, thereby protecting the liver from I/R damage^34^. Hyperglycemia not only exacerbates I/R injury, but it also eliminates or weakens the protective effect of various interventions on organs after I/R. Wang et al.^35^ established a rat model of acute hyperglycemia via intraperitoneal injection of 25% glucose 45 min before renal ischemia. Their study found that hyperglycemia not only aggravated RI/RI, but also eliminated the protective effect of dexmedetomidine pretreatment on renal tissue in rats^35^. Gao et al.^29^ demonstrated that ischemic post-conditioning with sevoflurane was effective in preventing myocardial I/R injury in normal rats, but not in diabetic rats, which may be associated with the impaired Nrf2/HO-1 signaling pathway in the myocardial cells of diabetic rats. Both *in-vitro* and *in-vivo* experiments have shown that isoflurane lost its protective effects on the heart during cardiac I/R injury under a high glucose environment, which may be associated with inhibition of Nrf2 signaling^30^. However, Zhang et al.^5^ revealed that sevoflurane protected the kidney against I/R injury through the reduction in the expression of NF-κB and TNF-α in the renal tissues of diabetic rats. In this study, we found that IsoP can improve renal function, mitigate renal tubular damage and apoptosis, and reduce oxidative stress and inflammation in diabetic rats subjected to I/R, which is accompanied by a decreased NF-κB level and the increased expression of Brg1, Nrf2, and HO-1. Taken together, these results suggest that IsoP can alleviate RI/RI via enhancing the antioxidant power of the Brg1/Nrf2/HO-1 signaling pathway under high glucose conditions.

The medication modes of inhaled anesthetics in surgery related to RI/RI are divided into pre-administration, total administration and post-administration, all of which can achieve the renal protective effect of isoflurane. With the development of anesthesiology in recent years, pre-treatment administration has been increasingly used in clinical anesthesia, especially for renal failure patients, due to its advantages of achieving the expected anesthetic effect at a lower dose, preventing drug accumulation and improving patient comfort. In our study, we examined only a single concentration (2%, a dose within the range of clinical use) for a single duration (30 min) of isoflurane preconditioning. Therefore, whether the renal protective effect of isoflurane is dose-dependent and time-dependent, it cannot be determined from this study, which requires further research in the future.

## Conclusion

Our study provides evidence that isoflurane can exert a renoprotective effect against I/R-induced kidney damage in diabetic rats through the activation of Brg1/Nrf2/HO-1 signaling. Therefore, isoflurane could be regarded as a potential therapeutic agent to relieve RI/RI in patients with diabetes.

## Data Availability

Data sharing is not applicable.

## References

[1] Ponticelli C. (2014). Ischaemia-reperfusion injury: a major protagonist in kidney transplantation. Nephrol Dial Transplant.

[2] Fung A, Zhao H, Yang B, Lian Q, Ma D (2016). Ischaemic and inflammatory injury in renal graft from brain death donation: an update review. J Anesth.

[3] MacIsaac RJ, Jerums G, Ekinci EI (2018). Glycemic Control as Primary Prevention for Diabetic Kidney Disease. Adv Chronic Kidney Dis.

[4] Xiao YD, Huang YY, Wang HX, Wu Y, Leng Y, Liu M, Sun Q, Xia ZY (2016). Thioredoxin-Interacting Protein Mediates NLRP3 Inflammasome Activation Involved in the Susceptibility to Ischemic Acute Kidney Injury in Diabetes. Oxid Med Cell Longev.

[5] Zhang Y, Hu F, Wen J, Wei X, Zeng Y, Sun Y, Luo S, Sun L (2017). Effects of sevoflurane on NF-small ka, CyrillicB and TNF-alpha expression in renal ischemia-reperfusion diabetic rats. Inflamm Res.

[6] Yan W, Chen Z, Chen J, Chen H (2016). Isoflurane preconditioning protects rat brain from ischemia reperfusion injury via up-regulating the HIF-1alpha expression through Akt/mTOR/s6K activation. Cell Mol Biol (Noisy-le-grand).

[7] Zhou Y, Peng DD, Chong H, Zheng SQ, Zhu F, Wang G. (2019). Effect of isoflurane on myocardial ischemia-reperfusion injury through the p38 MAPK signaling pathway. Eur Rev Med Pharmacol Sci.

[8] Li X (2020). Isoflurane suppresses lung ischemia-reperfusion injury by inactivating NF-kappaB and inhibiting cell apoptosis. Exp Ther Med.

[9] Wang H, Guo L, Wang Y, Song S (2021). Isoflurane upregulates microRNA-9-3p to protect rats from hepatic ischemia-reperfusion injury through inhibiting fibronectin type III domain containing 3B. Cell Cycle.

[10] Liang Y, Li Z, Mo N, Li M, Zhuang Z, Wang J, Wang Y, Guo X (2014). Isoflurane preconditioning ameliorates renal ischemia-reperfusion injury through antiinflammatory and antiapoptotic actions in rats. Biol Pharm Bull.

[11] Qin Z, Lv E, Zhan L, Xing X, Jiang J, Zhang M (2014). Intravenous pretreatment with emulsified isoflurane preconditioning protects kidneys against ischemia/reperfusion injury in rats. BMC Anesthesiol.

[12] Trotter KW, Archer TK (2008). The BRG1 transcriptional coregulator. Nucl Recept Signal.

[13] Trotter KW, Archer TK (2007). Nuclear receptors and chromatin remodeling machinery. Mol Cell Endocrinol.

[14] Singh AP, Foley JF, Rubino M, Boyle MC, Tandon A, Shah R, Archer TK (2016). Brg1 Enables Rapid Growth of the Early Embryo by Suppressing Genes That Regulate Apoptosis and Cell Growth Arrest. Mol Cell Biol.

[15] Wu Wu, Madany P, Dobson JR, Schnabl JM, Sharma S, Smith TC, van Wijnen AJ, Stein JL, Lian JB, Stein GS, Muthuswami R, Imbalzano AN, Nickerson JA (2016). The BRG1 chromatin remodeling enzyme links cancer cell metabolism and proliferation. Oncotarget.

[16] Marathe HG, Watkins-Chow DE, Weider M, Hoffmann A, Mehta G, Trivedi A, Aras S, Basuroy T, Mehrotra A, Bennett DC, Wegner M, Pavan WJ, de la Serna IL (2017). BRG1 interacts with SOX10 to establish the melanocyte lineage and to promote differentiation. Nucleic Acids Res.

[17] Ge M, Yao W, Yuan D, Zhou S, Chen X, Zhang Y, Li H, Xia Z, Hei Z (2017). Brg1-mediated Nrf2/HO-1 pathway activation alleviates hepatic ischemia-reperfusion injury. Cell Death Dis.

[18] Li Y, Zhao Y, Cheng M, Qiao Y, Wang Y, Xiong W, Yue W (2018). Suppression of microRNA-144-3p attenuates oxygen-glucose deprivation/reoxygenation-induced neuronal injury by promoting Brg1/Nrf2/ARE signaling. J Biochem Mol Toxicol.

[19] Zhang J, Ohta T, Maruyama A, Hosoya T, Nishikawa K, Maher JM, Shibahara S, Itoh K, Yamamoto M (2006). BRG1 interacts with Nrf2 to selectively mediate HO-1 induction in response to oxidative stress. Mol Cell Biol.

[20] Gong DJ, Wang L, Yang YY, Zhang JJ, Liu XH (2019). Diabetes aggravates renal ischemia and reperfusion injury in rats by exacerbating oxidative stress, inflammation, and apoptosis. Ren Fail.

[21] Jablonski P, Howden BO, Rae DA, Birrell CS, Marshall VC, Tange J (1983). An experimental model for assessment of renal recovery from warm ischemia. Transplantation.

[22] Forini F, Nicolini G, Kusmic C, Iervasi G (2019). Protective Effects of Euthyroidism Restoration on Mitochondria Function and Quality Control in Cardiac Pathophysiology. Int J Mol Sci.

[23] Kolachala VL, Palle SK, Shenoi A, Shayakhmetov DM, Gupta NA (2019). Influence of Fat on Differential Receptor Interacting Serine/Threonine Protein Kinase 1 Activity Leading to Apoptotic Cell Death in Murine Liver Ischemia Reperfusion Injury Through Caspase 8. Hepatol Commun.

[24] Shi S, Lei S, Tang C, Wang K, Xia Z (2019). Melatonin attenuates acute kidney ischemia/reperfusion injury in diabetic rats by activation of the SIRT1/Nrf2/HO-1 signaling pathway. Biosci Rep.

[25] Ishii T, Mann GE (2014). Redox status in mammalian cells and stem cells during culture in vitro: critical roles of Nrf2 and cystine transporter activity in the maintenance of redox balance. Redox Biol.

[26] Gold R, Kappos L, Arnold DL, Bar-Or A, Giovannoni G, Selmaj K, Tornatore C, Sweetser MT, Yang M, Sheikh SI, Dawson KT, Investigators DS (2012). Placebo-controlled phase 3 study of oral BG-12 for relapsing multiple sclerosis. N Engl J Med.

[27] Chen HH, Lu PJ, Chen BR, Hsiao M, Ho WY, Tseng CJ (2015). Heme oxygenase-1 ameliorates kidney ischemia-reperfusion injury in mice through extracellular signal-regulated kinase 1/2-enhanced tubular epithelium proliferation. Biochim Biophys Acta.

[28] Holmstrom KM, Kostov RV, Dinkova-Kostova AT (2016). The multifaceted role of Nrf2 in mitochondrial function. Curr Opin Toxicol.

[29] Gao S, Yang Z, Shi R, Xu D, Li H, Xia Z, Wu QP, Yao S, Wang T, Yuan S (2016). Diabetes blocks the cardioprotective effects of sevoflurane postconditioning by impairing Nrf2/Brg1/HO-1 signaling. Eur J Pharmacol.

[30] Wang Y, Li H, Huang H, Liu S, Mao X, Wang S, Wong SS, Xia Z, Irwin MG (2016). Cardioprotection from emulsified isoflurane postconditioning is lost in rats with streptozotocin-induced diabetes due to the impairment of Brg1/Nrf2/STAT3 signalling. Clin Sci (Lond).

[31] Leung JM, Goehner P, O’Kelly BF, Hollenberg M, Pineda N, Cason BA, Mangano DT (1991). Isoflurane anesthesia and myocardial ischemia: comparative risk versus sufentanil anesthesia in patients undergoing coronary artery bypass graft surgery. The SPI (Study of Perioperative Ischemia) Research Group. Anesthesiology.

[32] Ran K, Zou DQ, Xiao YY, Chang YT, Duan KM, Ou YW, Li ZJ (2015). Effects of isoflurane preconditioning in the delayed phase on myocardial tumor necrosis factor alpha levels and caspase-3 protein expression in a rabbit model of ischemia-reperfusion injury. Genet Mol Res.

[33] Peng Z, Ban K, Wawrose RA, Gover AG, Kozar RA (2015). Protection by enteral glutamine is mediated by intestinal epithelial cell peroxisome proliferator-activated receptor-gamma during intestinal ischemia/reperfusion. Shock.

[34] Yang P, Du Y, Zeng H, Tian C, Zou X. (2019). Comparison of Inflammatory Markers Between the Sevoflurane and Isoflurane Anesthesia in a Rat Model of Liver Ischemia/Reperfusion Injury. Transplant Proc.

[35] Wang H, Chen H, Wang L, Liu L, Wang M, Liu X (2014). Acute hyperglycemia prevents dexmedetomidine-induced preconditioning against renal ischemia-reperfusion injury. Acta Cir Bras.

